# A Protocol for Organoids from the Urine of Bladder Cancer Patients

**DOI:** 10.3390/cells12172188

**Published:** 2023-08-31

**Authors:** Simon Walz, Paul Pollehne, Ruizhi Geng, Johannes Schneider, Moritz Maas, Wilhelm K. Aicher, Arnulf Stenzl, Bastian Amend, Niklas Harland

**Affiliations:** 1 Department of Urology, Tuebingen University Hospital, 72076 Tübingen, Germany; simon.walz@med.uni-tuebingen.de (S.W.); niklas.harland@med.uni-tuebingen.de (N.H.); 2 Center for Medical Research, University of Tuebingen, 72074 Tübingen, Germany

**Keywords:** bladder cancer, organoids, personalized medicine

## Abstract

This study investigates the feasibility of establishing urine-derived tumor organoids from bladder cancer (BC) patients as an alternative to tissue-derived organoids. BC is one of the most common cancers worldwide and current diagnostic methods involve invasive procedures. Here, we investigated the potential of using urine samples, which contain exfoliated tumor cells, to generate urine-derived BC organoids (uBCOs). Urine samples from 29 BC patients were collected and cells were isolated and cultured in a three-dimensional matrix. The establishment and primary expansion of uBCOs were successful in 83% of the specimens investigated. The culturing efficiency of uBCOs was comparable to cancer tissue-derived organoids. Immunohistochemistry and immunofluorescence to characterize the uBCOs exhibited similar expressions of BC markers compared to the parental tumor. These findings suggest that urine-derived BC organoids hold promise as a non-invasive tool for studying BC and evaluating therapeutic responses. This approach could potentially minimize the need for invasive procedures and provide a platform for personalized drug screening. Further research in this area may lead to improved diagnostic and treatment strategies for BC patients.

## 1. Introduction

With more than 570,000 newly diagnosed cases worldwide, bladder cancer (BC) accounts for one of the most common cancers in men and women in general [1]. Primary diagnostics in BC assessment include urine cytology, as BC grows in direct contact with the urinary tract and releases exfoliated cells into the voiding urine, as well as invasive cystoscopic examination and resection followed by pathological evaluation of the tissue. Treatment regimens for non-muscle-invasive bladder cancer (NMIBC) and muscle-invasive bladder cancer (MIBC) differ. Whereas NMIBC, which accounts for 75% of the newly diagnosed cases, is treated with a transurethral removal of bladder tumor (TURBT), frequently followed by intravesical instillation therapy depending on the risk-stratification, MIBC is commonly treated with a neoadjuvant systemic approach followed by radical cystectomy and urinary diversion [2]. In NMIBC, approximately 70% of patients experience recurrent disease, and around 10–15% of patients progress to MIBC [3]. Following curative intended treatment for MIBC, local recurrence is observed in only 5–15% of patients, whereas distant spread of the disease occurs in 50% of patients, primarily within the first 2 years post-treatment [4,5]. In metastatic urothelial cancer, systemic therapies with cytotoxic and immunological agents, as well as antibody-drug conjugates, are commonly used. Response rates in the neoadjuvant and the metastatic setting differ, and predictive markers for sufficient treatment response and individualized therapeutic approach are rare [6,7].

In recent times, three-dimensional bladder cancer organoids (BCO), derived from tumorous bladder cancer tissue (tBCO), were introduced into uro-oncological research. Several years ago, organoids were defined as three-dimensional in vitro cell culture constructs which contain cells colonizing on a porous matrix and hold the capability to generate tumor-like structures [8,9,10]. In addition, organoids also host epidermal and endothelial cells either provided ex vivo as by-catch with the maternal specimen or after in vitro differentiation of progenitor cells, as well as cells of mesenchymal origin [9,11]. Based on the heterogeneity and the structure of the BCO itself, three-dimensional BCOs show a more realistic simulation of the actual tumor architecture and environment compared to two-dimensional cell cultures [10,11,12]. 

BCOs hold the potential to facilitate drug development and lay the basis for an individualized multi-drug screening tool, as the histological and molecular spectrum of the maternal tumor is also represented within its derived organoids [10,13,14]. In recent approaches in BC and other entities, patient-derived organoids were used to evaluate the therapeutic sensitivity of several drugs and displayed consistent results regarding embedded genomic alterations [13,15,16,17,18,19,20]. 

One of the major disadvantages of the establishment of patient-derived BCOs is displayed in the need for an invasive procedure (TURBT, nephroureterectomy, or cystectomy) to harvest the malignant cells from the bladder to sow the organoids. Since BC exfoliates tumor cells into the patient’s urine, which is commonly used as a diagnostic tool in urine cytology, fluorescence in situ hybridization, nuclear matrix protein-22, and many others should also hold the potential to generate urine-derived bladder cancer organoids (uBCO) to spare the invasive procedure and thereby minimize the risk for the patient [21,22,23]. Recently, in a first feasibility attempt, uBCOs of MIBC in a canine model were successfully established. The authors reported sufficient uBCO efficiency and provided a platform for in vitro drug screening for canine BC therapy [24]. 

In this study, we evaluated the feasibility of establishing urine-derived tumor organoids directly from rinsing urine samples of BC patients without any intermediate host and compared the efficiency to tBCOs. We were able to establish a long-living urine-derived tumor line in a three-dimensional scaffold and found a comparable culturing efficiency for urine-derived and tissue-derived organoids. In addition, urine-derived cancer organoids show similar expression levels of antigens typical for BC, as well as tumor stem-cell markers when compared to their maternal tumor. 

## 2. Materials and Methods

### 2.1. Isolation of Cells from the Urine of Bladder Cancer Patients and Expansion of Cells in Organoid Cultures

**Figure 1:**
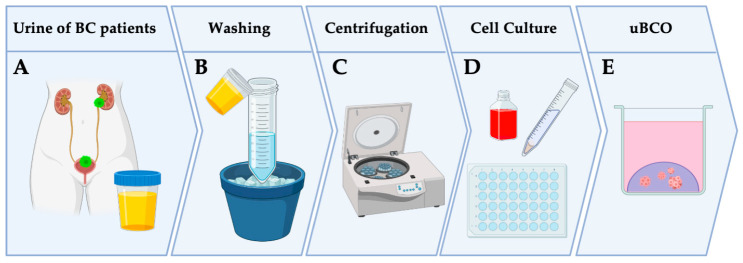
Method and workflow of the culturing process of uBCOs. The materials and methods section contains a comprehensive and detailed step-by-step workflow, along with the materials and reagents used in the study. Abbreviations: BC, bladder cancer; uBCO, urine-derived bladder cancer organoid. Created with biorender.com (accessed 12 May 2023).

Rinsing urine collected prior to TURBT was buffered immediately after collection with an equal volume of cold PBS (Dulbecco’s Phosphate Buffered Saline, Gibco Life Technologies, Carlsbad, CA, USA) and refrigerated at 4 °C on wet ice until processing (Figure 1A). Urine samples were processed within a maximum of 4 h from the time of collection.

Urine samples were transferred to sterile 50 mL centrifugation tubes and centrifuged at 250 g for 10 min at room temperature (RT) (Centrifuge Heraeus Multifuge 3SR+, Thermo Fisher Scientific, Osterode, Germany). The urine supernatant was removed to 1 mL and the tissue pellet was resuspended, transferred to a tube, and washed with washing buffer (Figure 1B; 98.8 mL Dulbecco’s Phosphate Buffered Saline, Gibco Life Technologies, Carlsbad, CA, USA; 1 mL Penicillin/Streptomycin 10,000 i.E./mL, Thermo Fischer, Waltham, MA, USA; 0.2 mL Amphotericin B, Sigma Aldrich, Taufkirchen, Germany). Again, the supernatant was removed after centrifugation at 250 g for 10 min at RT (Figure 1C), the pellet was resuspended followed by cell counting in the Neubauer improved counting chamber (Neubauer improved counting chamber 0.1 mm depth, 0.0025 mm^3^, Assistant Germany, Sonderheim, Germany; Mikroskop Axiovert 135, Zeiss Germany, Jena, Germany), and the cell count of the sample was adjusted to 2 × 10^6^ cells/mL by adding media or by further centrifugation and resuspension in less media. Then, cells were mixed on wet ice with three times the volume of base membrane extract (Matrize BME Typ2 Cultrex^®^, R&D Systems, Minneapolis, MN, USA; 3:1 in working buffer (44.5 mL Advanced DMEM/F12, Thermo Fischer, Waltham, MA, USA; 5 mL Fetal Bovine Serum, Sigma Aldrich, Taufkirchen, Germany; 0.5 mL Penicillin/Streptomycin 10,000 i.E./mL, Thermo Fischer, Waltham, MA, USA), resuspended carefully, and plated out on 2–7 wells of a 48-well plate (48 well cell culture plate Costar^®^, Corning Inc., Kennbunk, ME, USA) as described recently (Figure 1D) [25]. If very few or no cells were visible during cell counting, the sample was centrifuged and the supernatant was removed to 10 µL, resuspended with 30 µL of BME on ice, and plated out on 2 wells of a 48-well plate as a droplet. A 20 µL aliquot of the cell-BME suspension was used for each well, which formed a hemispherical three-dimensional structure on the bottom of the wells and are further referred to as domes.

The 48-well plate was inverted and incubated at 37 °C and 5% CO_2_ for 15 min, and then each well was covered with 250 µL of bladder tumor medium ((BTM): 22.5 mL L-WRN conditioned medium, in-house product; 22 mL Advanced DMEM(1×)/F-12, Thermo Fischer, Waltham, MA, USA; 2.5 mL of 5% FBS, Sigma Aldrich, Taufkirchen, Germany; 1 mL B27 supplement, Gibco Life Technologies, Carlsbad, CA, USA; 500 μL L-glutamine, Biochrom, Berlin, Germany; 500 μL HEPES 1 M, Gibco Life Technologies, Carlsbad, CA, USA; 500 μL nicotinamide 1 M, Sigma Aldrich, Taufkirchen, Germany; 125 μL N-acetylcycsteine 500 mM, Sigma Aldrich, Taufkirchen, Germany; 50 μL A83-01 5 mM Tocris Bioscience, Bristol, UK; 50 μL Primocin 50 mg/mL, Thermo Fischer, Waltham, MA, USA; 50 μL FGF-10 100 μg/mL, Peprotech GmbH, Cranbury, NJ, USA; 25 μL FGF-7 50 μg/mL, Peprotech GmbH, Cranbury, NJ, USA; 12.5 μL FGF-2 50 μg/mL, Peprotech GmbH, Cranbury, NJ, USA; 5 μL Y-27632 100 mM, Hycultec, Beutelsbach, Germany, and 0.5 μL EGF 500 μM, Peprotech GmbH, Cranbury, NJ, USA). Representative domes were captured as light microscopy images and the cell culture was then incubated at 37 °C and 5% CO_2_. 

The cultured bladder tumor cells were evaluated by light microscopy (Axiovert 135, Zeiss Germany, Jena, Germany) at intervals of 1–3 days and assessed regarding their size increase and the morphology of the cultured cells/cell conglomerates (Figure 1E).

The urine and tumor cell cultures were passaged when they showed strong growth of organoids and the supply of BTM, or when the integrity of the cast dome appeared to be at risk. Cells were also passaged when light microscopy showed the secretion of membrane vesicles as a sign of impending apoptosis. The splitting ratio was determined based on growth behavior and the estimated number of cells in the wells (Figure 1).

### 2.2. Isolation of Cells from Urothelial Carcinoma Tissue from Bladder Cancer Patients and Expansion of Cells in Organoid Cultures

The culturing of tBCOs was performed according to the previously published protocol [25]. In summary, the tissue was minced and collected using centrifugation at 480 g for 10 min at RT (Centrifuge Heraeus Multifuge 3SR+, Thermo Fisher Scientific, Osterode, Germany). The sediment obtained was resuspended in a buffer containing 3000 U/mL collagenase and 1000 U/mL hyaluronidase (10×, Stem Cell Technologies, Vancouver, BC, Canada) and incubated with moderate agitation at 37 °C for 30 min. Proteolytic degradation was continued by adding fresh collagenase and incubating for an additional 30 min at 37 °C. Debris was removed using a cell strainer (70 μm mesh; BD Bioscience Discovery Labware, Bedford, MA, USA) and the filtrate was centrifuged at 150 g for 7 min at RT. The resulting cells’ yield and viability were assessed and then resuspended at 4 × 10^6^/mL to obtain 4 × 10^4^ cells in 10 μL. Next, these cells were mixed with 30 μL basal membrane extract (Matrize BME Typ2 Cultrex^®^, R&D Systems, Minneapolis, MN, USA) on ice, transferred to a 24-well plate (Multiwellplatte, Greiner Bio-one GmbH, Frickenhausen, Germany), and inverted to create hanging drops. After a short incubation at 37 °C, the plates were flipped back and supplemented with 500 μL of BTM culture medium per well. The plates were then incubated in a cell-culture incubator (37 °C, 5% CO_2_, humidified atmosphere; Inkubator CB120, Binder, Tuttlingen, Germany). All organoid cultures included in the study were downscaled to fit 48-well plates (48 well cell culture plate Costar^®^, Corning Inc., Kennbunk, ME, USA) [26].

### 2.3. Immunohistochemistry and Immunofluorescence of Organoids

To characterize the tumor organoids, two distinct methods of immunohistochemistry of paraffin sections and immunofluorescence were applied.

Immunohistochemistry was utilized to detect the expression of the GATA-3, CK5/6, CK7, p53, p63, CD24, and CD44. The primary antibodies were washed off (3× for 5 min, PBS), and detected by the HRP or AP polymer reagent (IHC ZytochemPlus). The samples were counterstained by HE, covered (VectaMount, Vectorlabs), and recorded by microscopy (Axiovert A1, Zeiss) as described [25].

For three-dimensional evaluation of the uBCOs, organoids were cultured in 8-well chamber slides. Immediately prior to staining, 100 µL dispase (Dispase II, Merck, Darmstadt, Germany) was added to the medium. After incubation, the uBCOs were centrifuged and the supernatant was discarded. The uBCOs were fixed by 4% formaldehyde (30 min, RT), washed well three times with PBS, blocked (5% BSA, 0.2% Triton X-100, 0.1% Tween 20, in PBS; 1 h, RT), and incubated (1 h, 37 °C, humidified chamber, dark) with primary antibodies to GATA-3, AE1/AE3, CK5/6, CK7, CK20, p53, TP63, Vimentin, s100P, CD24, CD44, Ki67, and FGFR3 at optimized concentrations (Tbl.S.1). Unbound primary antibodies were washed away (3 × 5 min, PBS, RT). Primary antibodies were detected by incubation of the samples with complementary fluorescence-labeled secondary antibodies (1 h, RT, humidified chamber, dark) as requested by the supplier. Unbound secondary antibodies were rinsed away (3 × 5 min, PBS, RT). Cellular nuclei were counterstained by DAPI and the expression of the marker genes was visualized by microscopy (Leica Stellaris 8 or Zeiss Axiophot). The antibody diluent was 1% BSA in PBS. Samples omitting the primary antibodies and samples stained with mouse or rabbit IgG isotype antibodies served as controls [25].

### 2.4. Software and Statistics

Data are displayed as median if applicable and box plots are displayed as median with 25th or 75th quantiles and min/max whiskers. Individual groups were tested using the Mann–Whitney U-test. Spearman’s rho (ρ) was calculated for correlation between continuous data. A two-sided Fisher exact test was used to evaluate the differences in efficiency rates. Graphs were plotted using GraphPad Prism v.8.4.0. Statistical analyses were conducted using GraphPad Prism v.9.4.1 and JMP (SAS Institute, v.16.0.0) software. *p* values < 0.05 were considered statistically significant. The workflow was created using biorender.com (accessed 12 May 2023).

## 3. Results

### 3.1. Patient Characteristics

Urine samples from a total of 29 patients with pathologically confirmed, active urothelial cancer at primary diagnosis or relapse were included in this study. In 13/29 patients (45%), concomitant tumor tissue was collected for the establishment of additional tBCOs. The median age of the patients at the sample collection was 66 years (range: 30–85). Among the patients, 83% were male. The majority of patients (69%) had NMIBC and 59% showed high-grade histopathological features. A total of 72% of patients had positive urine cytology at the time of sample collection. Tumor-specific treatment was applied in 45% of patients prior to sample collection, with local surgery (34%) or instillation (7%) therapies applied most commonly. Detailed patient characteristics are shown in Table 1.

### 3.2. Efficient Culturing of Urine-Derived Bladder Cancer Organoids

For the establishment of uBCOs cells from a median of 280 mL (100–400 mL), rinsing urine was cultured in a three-dimensional matrix after centrifugation and washing (Figure 1).

**Figure 2:**
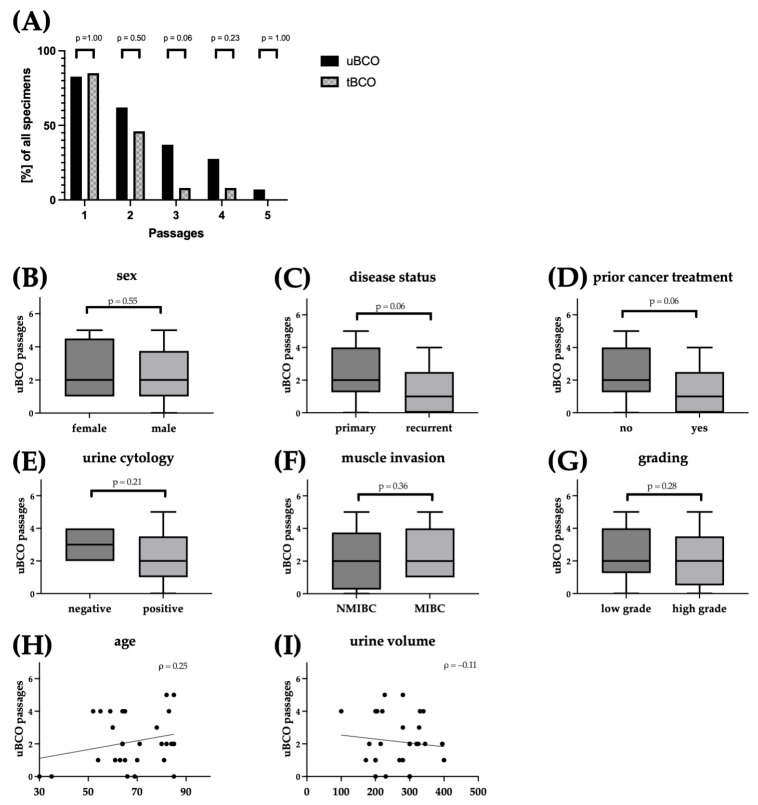
Culturing efficiency of uBCOs (indicated in black) and tBCOs (indicated in grey hatched) (**A**) with respect to the individual passages (efficiency rate). Culturing efficiency according to patient characteristics and histopathological features with regard to the maximum achieved passage (**B**–**I**). Dots represent data from individual organoids. Boxes indicate median and 25th and 75th percentiles with min/max whiskers. *p*-values indicate differences between two groups (A: two-sided Fischer exact test; B: Mann–Whitney-U test). The ρ-values indicate linear correlations between two parameters (Spearman correlation). Abbreviations: uBCO, urine-derived bladder cancer organoid; tBCO, tissue-derived bladder cancer organoid; NMIBC, non-muscle- invasive bladder cancer; MIBC, muscle-invasive bladder cancer.

Cultures of uBCOs were successfully established for 83% of specimens in the first passage. The subsequent expansion of uBCOs was achieved for 62% of samples, with 7% reaching the fifth passage (Figure 2A). The culturing efficiency for uBCOs was independent of patients’ sex (*p* = 0.55; Figure 2B), disease status (*p* = 0.06; Figure 2C), prior cancer-specific treatment (*p* = 0.06; Figure 2D), urine cytology (*p* = 0.21; Figure 2E), or histopathological features such as muscle invasion (*p* = 0.36; Figure 2F) and grading (*p* = 0.28; Figure 2G). Age (ρ = 0.25; Figure 2H) and, of note, the volume of rinsing urine (ρ = 0.11; Figure 2I) showed no linear correlation to the culturing efficiency of uBCOs. 

Corresponding tBCOs were established based on previously described protocols [13,25,26]. Culturing efficiency of uBCO was comparable or even increased compared to tBCOs, with 83% and 85% (*p* = 1.00), 62% and 46% (*p* = 0.50), 37% and 8% (*p* = 0.06), 28% and 7% (*p* = 0.23), and 7% and 0% (*p* = 1.00) for passages 1 to 5, respectively (Figure 2I).

Comparative analyses of mean organoid diameters from paired specimens at two consecutive time points during passage 1 showed individual growth behavior. In detail, both an interindividual faster increase of the diameter for uBCO and tBCO, respectively, and almost parallel behavior were detected (Figure S1). Semi-quantitative analysis revealed an increase in organoid cell mass (well count Δ from primary culture to passage 1) in 5/11 (45%) of uBCO specimens and 7/11 (63%) of tBCO specimens, with a higher mean Δ of organoid cell mass for growing uBCOs compared to growing tBCOs (mean 40% vs. 17%) (Table 2).

### 3.3. Urine-Derived Bladder Cancer Organoids Show Classical Features of Organoids and Mimic Primary Bladder Cancer

**Figure 3:**
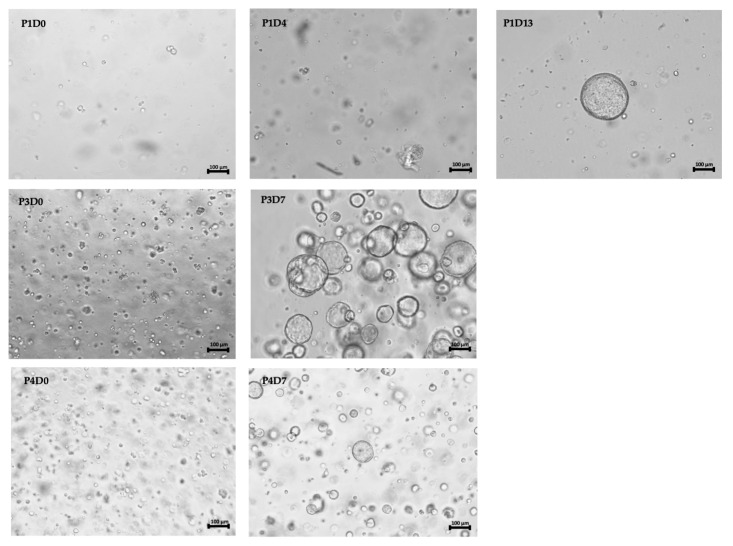
Light microscopic images of uBCO #027 during cultivation in basal membrane extract in the upper row on the first, fourth, and thirteenth day of the first passage, in the middle and lower row on the first and seventh day of the third and fourth passage, respectively. Size bars indicate 100 mm. Abbreviations: uBCO, urine-derived bladder cancer organoid; P, passage; D, day.

Light microscopy of expanding uBCO showed classical features of tissue-derived organoids, with cell clustering and subsequent organoid formation. Organoid formation and expansion occurred early in higher passages (Figure 3). Further characterization of exemplary uBCOs (*n* = 6) was performed using immunohistochemistry and multidimensional immunofluorescence. The uBCOs showed, in general, positivity for markers of urothelial differentiation (GATA-3, S100P), cytokeratins typically expressed in BC (AE1/AE3, CK5, CK 7, CK20), and for the proliferation marker Ki67. TP63 and p53 showed positivity in 67% and 100% uBCOs, respectively. A total of 50% of uBCO showed mesenchymal differentiation with positivity for vimentin. Immune checkpoint antigen CD276, also known as B7-H3, and stemness markers, the sialoglycoprotein CD24, and cell-surface glycoprotein CD44 showed positivity in 67%, 33%, and 67%, respectively (Table 3). None of the investigated uBCOs showed an expression of FGFR3. The detailed immunofluorescence characterization of several uBCOs is provided in Supplement Figure S2.

**Figure 4:**
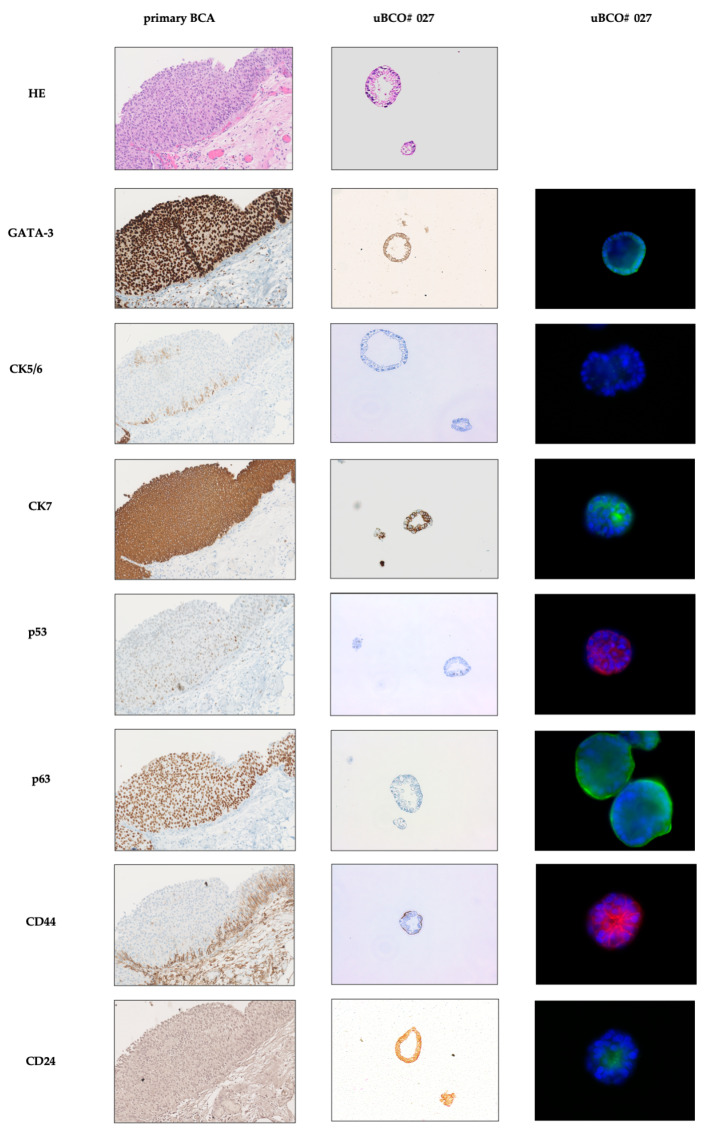
Immunohistochemical images from parental primary BC (left column) and autologous, uBCO #027 (middle column) in the fifth passage. The left column displays fluorescence staining of relevant antigens in urothelial cancer or tumor stem cell antigens in the corresponding uBCO #027 (passage 5). The tBCO only reached the first passage and could, therefore, not be implemented in the analysis. Abbreviations: BC, bladder cancer; uBCO, urine-derived bladder cancer organoid; tBCO, tissue-derived bladder cancer organoid HE, hematoxylin-eosin; GATA, glutamyl amino tranverase A; CK, cytoceratine; p, protein; CD, cluster of differentiation.

A direct comparison of immunohistochemistry and immunofluorescence markers in uBCO and parental primary tumor revealed comparable expressions (Figure 4). The impaired growth of the paired tBCO prevented the direct comparison, as it only reached the first passage. 

## 4. Discussion

Patient-derived organoids have emerged as a promising tool in oncological research, allowing for the cultivation of three-dimensional tumor models that closely mimic the histological and molecular characteristics of the parental tumor [17,27]. However, the efficient cultivation of organoids is often hampered by the lack of sufficient vital primary tumor tissue. In recent years, multiple approaches have been developed to use urine as a tool for diagnostics, characterization, and monitoring of BC [21,22,23]. In particular, the presence of vital tumor cells in urine samples of BC patients, as obvious in routinely applied urine cytology, emphasizes the potential of urine as a basis for the establishment of patient-individual organoids [23]. In this study, we report a protocol for the establishment of urine-derived tumor organoids as a non-invasive alternative to tissue-derived organoids. 

We present evidence of the effective generation of uBCOs directly from the rinsing urine of BC patients. In line with previous reports, successful organoid establishment was defined based on morphological and immunohistochemical features independent of the number of passages reached [17]. This finding is significant, as it indicates that urine samples, which can routinely be collected for diagnostic purposes, can serve as a valuable source for generating BCOs, eliminating the need for invasive procedures such as TURBT, nephroureterectomy, or cystectomy.

For the expansion of urine-derived cells in uBCOs, the media and procedures published recently were employed [26]. In an independent study, we compared the culturing efficacy of tBCOs upon expansion following two different published protocols [13,26]. Neither protocol nor procedure was superior to the other. Some tBCOs were even expanded in one of the two media and then changed to the other with no difference in proliferation or appearance of the organoids, whereas others stopped proliferation upon media change. This occurred both ways. Moreover, a pattern of media preference was not recorded at all (unpublished observation). In another study, requirements for individual growth factors or other low molecular weight compounds for the expansion of spheroids generated from bladder cancer cell lines, as well as from primary cells prepared from BC tumor samples, were investigated. Again, a clear trend towards an individual factor was not observed (unpublished observation). Therefore, we refrained from transferring such studies to uBCO productions. Though the processes of culturing uBCOs and tBCOs share similarities, there are significant differences in the preparation of the primary tumor cells used. The isolation of tumor cells from the urine of BC patients primarily relies on centrifugation and washing steps. In contrast, preparing primary tumor tissue involves mechanical mincing followed by proteolytic degradation before introducing the cells into the culturing process. The chance to mitigate the impact of proteolytic degradation on the introduced tumor cells in uBCOs could potentially enhance the comparability between parental tumors and derived uBCOs compared to tBCOs. This aspect holds significant interest for future studies, although it could not be implemented in this feasibility study due to the challenge of achieving parallel growth for both uBCOs and tBCOs. 

The culturing efficiency of uBCO was found to be similar to the rates of successful organoid formations in a canine urinary-derived BCO model [24]. Compared to the culturing efficiency of autologous tBCO, rinsing urine as starting material for uBCOs showed at least an equivalent rate of organoid formation. Though the culturing efficiency of uBCOs in early passages was comparable to recent reports on tBCOs, long-living uBCOs were scarce compared to tBCOs in the current literature [26]. This might be due to an individual, limited expansion capacity of the implemented cells, as the autologous comparison of u- and tBCO culturing efficiency showed a concordant behavior within our study. The independence of culturing efficiency from various patient and disease characteristics, consistent with prior findings on tBCOs, as demonstrated by Lee et al., serves to strengthen the feasibility of this approach [13]. Specifically, the culture efficiency did not show a statistically significant decrease in patients with prior drug therapy (*p* = 0.06) and in cases of low-grade carcinomas (*p* = 0.06). However, it is important to reevaluate this trend in a larger group of patients to obtain more conclusive results.

Light microscopy revealed a comparable morphology and three-dimensional architecture of uBCOs and tBCOs, as well as reported appearances of BCOs [26,27]. The immunohistochemical evaluation of uBCOs exposed similar expressions typical for BC, such as GATA-3, S100P, and various cytokeratins in detail, CK5, CK 7, and CK20 [28,29,30]. As earlier described for tBCOs, the expression patterns of the parental tumor were found to be preserved in the derived uBCO [17,27]. Additionally, uBCOs exhibited the expression of designated tumor stem-cell markers, in detail, CD24 and CD44, further highlighting their potential for studying BC stem cells and their capacity to expansion, as well as their responses to systemic anti-cancer agents in BC [31]. 

The establishment of uBCOs offers distinct advantages over tBCOs. In this feasibility trial, rinsing urine was utilized to simultaneously acquire comparable tissue samples alongside subsequent TURBT procedures. Going forward, the focus should shift to employing voiding urine samples to enhance patient comfort; reduce invasiveness; and, subsequently, lower potential risks. Furthermore, uBCOs could serve as a foundation for drug testing and the exploration of personalized therapeutic strategies, as they faithfully reflect the phenotypical attributes of the primary tumor. The approach of using voiding urine would also enable the creation of uBCOs in parallel with the treatment timeline, presenting a real-time ex vivo/in vitro screening tool. Further trials are imperative to investigate the feasibility of utilizing voiding urine instead of rinsing urine for uBCO generation.

Although the results hold promise, it is important to acknowledge several limitations. The trial’s feasibility-oriented design did not allow for investigating specific cell populations, such as immune cells, in both the parental tumor and the generated uBCOs or tBCOs. Though the comparison between the parental tumor and its derived uBCOs could have been offered, the limitations imposed by the available materials hindered a simultaneous immunohistochemical or immunofluorescence characterization of both uBCOs and tBCOs in comparable, higher passages. Additionally, due to a relatively small cohort, it is imperative to validate these findings with a larger group of bladder cancer patients to ensure the reproducibility and broader applicability of the outcomes. Furthermore, though the evaluation of specific markers displayed consistency with the parental tumor tissue, a more comprehensive understanding of their similarity would necessitate further molecular characterization and a comparative analysis of genetic alterations between uBCOs, tBCOs, and primary bladder cancer tissue.

## 5. Conclusions

Taken together, this study demonstrates the feasibility and comparable efficiency of establishing uBCOs from rinsing urine samples of BC patients. These uBCOs faithfully recapitulate the histological and molecular features of BC and offer a non-invasive approach for personalized tumor modeling and individualized drug screening. Further research and validation are warranted to fully explore the potential of urine-derived organoids in BC research and clinical applications.

## Figures and Tables

**Table 1:** Patient characteristics *n* = 29; * patients suffered from concomitant CIS; abbreviations: TURBT, trans urethral removal of bladder tumor; CIS, carcinoma in situ; BCG, Bacillus Calmette Guerin; neoadj., neoadjuvant.

**Sex** (*n*)	
female	5
male	24
**Age** (years)	
Median	66
Range	(30–85)
**Urine volume** (mL)	
Median	280
Range	(172–400)
**Tissue acquisition**	
TURBT	28
Cystectomy	1
**Disease status**	
Primary diagnosis	16
Relapse	13
**Pathological features**	
CIS *	3
pTa	16
pT1	4
pT2	8
pT3	0
pT4	1
low grade	12
high grade	17
**Urine cytology**	
negative	4
positive	21
unkown	4
**Prior cancer treatment**	
non	16
TURBT	10
Mitomycin instillation	1
BCG instillation	1
neoadj. chemotherapy	1

**Table 2:** Number of wells in 48-well plates as a parameter of the expansion of cellular mass in primary culture and 1. passage of uBCO and tBCOs. Abbreviations: uBCO, urine-derived bladder cancer organoid; tBCO, tissue-derived bladder cancer organoid.

	Primary Culture (Wells)	1. Passage (Wells)	Δ (%)
uBCO #001	7	8	+14
tBCO #001	6	0	−100
uBCO #002	7	7	0
tBCO #002	6	7	+17
uBCO #003	7	7	0
tBCO #003	7	8	+14
uBCO #004	6	7	+17
tBCO #004	6	8	+33
uBCO #005	6	10	+66
tBCO #005	6	6	+17
uBCO #006	6	11	+83
tBCO #006	6	7	+17
uBCO #007	7	6	−14
tBCO #007	6	6	0
uBCO #008	26	0	−100
tBCO #008	3	0	−100
uBCO #009	5	7	+40
tBCO #009	7	3	−57
uBCO #010	29	7	−76
tBCO #010	6	21	+250
uBCO #011	28	21	−25
tBCO #011	6	9	+50

**Table 3:** Immunofluorescence characterization of the uBCOs in chamber slides and their corresponding exposure times. + displays a positive fluorescence signal, − indicates an absent fluorescence signal, and +/− displays a mixed fluorescence signal. Abbreviations: GATA, glutamyl amino transferase A; AE, anti-epithelial; p, protein; TP, tumor protein; CK, cytokeratin; CD, cluster of differentiation; uBCO, urine-derived bladder cancer organoid, Ki, Kiel; FGFR, fibroblast growth factor receptor; uBCO, urine-derived bladder cancer organoid; SS, shutter speed; ms, millisecond; n.a., not available.

	uBCO #013		uBCO #015		uBCO #019		uBCO #022		uBCO #027		uBCO #028	
		SS in ms		SS in ms		SS in ms		SS in ms		SS in ms		SS in ms
GATA-3	+	1638–2636	+/−	1000	+	1179–5000	+	1629–5083	+	668–1160	−	1000
AE1/AE3	+	363–524	+	108–1000	+	129–519	+	124–511	+	422–575	+	252–978
CK7	+	398–804	+	147–1000	+	177–217	+	150–199	+/−	210–859	+	258–1000
p53	+/−	3000	+/−	1000	+	3000	+	3489–4038	+/−	569–2000	+/−	1000
TP63	+	1792–3000	−	1000	+	308–3000	−	5000	+	2000	+/−	1000
Vimentin	+	1378–3000	+/−	1000	−	3000	−	5000	+	1589–2000	−	1000
S100P	−	3000	+/−	521–1000	+	114–423	+	571–1452	+	1624–2000	+	233–729
CK5	+	2076–3000	−	1000	−	3000	−	5000	+	2000	−	1000
CK20	−	3000	+/−	1000	+	302–1493	−	5000	−	2000	+	1000
CD276	−	3000	+/−	1000	+	981–3000	+	266–4714	+	747–859	−	1000
CD24	−	3000	−	1000	−	3000	+/−	579–5000	+/−	2000	−	1000
CD44	−	3000	+/−	1000	+/−	3000	−	3892–5000	+	179–500	+/−	386–402
Ki67		n.a.	+	1000	+	240–545	+	400–1000	+	353–911		n.a.
FGFR3	−	3000	−	1000	−	3000		n.a.	−	2000		n.a.

## Data Availability

The data of this study will be made available to all colleagues from public institution dedicated only to research and education upon justified request.

## Supplementary Materials

The following supporting information can be downloaded at: https://www.mdpi.com/article/10.3390/cells12172188/s1, Figure S1: Exemplarily shown, mean growth of three paired cultures of urin- (indicated in yellow) and tissue-derived (indicated in red) BCOs of the same patient displaying individual growth patterns within a time span of 5 days in the first passage; Figure S2: Immunofluorescence characterization of the uBCOs in chamber slides and their corresponding exposure times indicated in ms; Table S1: Antibodies employed for chamber slides and paraffin sections.

## References

[1] Sung H., Ferlay J., Siegel R.L., Laversanne M., Soerjomataram I., Jemal A., Bray F. (2021). Global Cancer Statistics 2020: GLOBOCAN Estimates of Incidence and Mortality Worldwide for 36 Cancers in 185 Countries. CA Cancer J. Clin..

[2] Walz S., Aslani V., Sawodny O., Stenzl A. (2023). Robotic radical cystectomy–more precision needed?. Curr. Opin. Urol..

[3] Soukup V., Capoun O., Cohen D., Hernandez V., Babjuk M., Burger M., Comperat E., Gontero P., Lam T., MacLennan S. (2017). Prognostic Performance and Reproducibility of the 1973 and 2004/2016 World Health Organization Grading Classification Systems in Non-muscle-invasive Bladder Cancer: A European Association of Urology Non-muscle Invasive Bladder Cancer Guidelines Panel Systematic Review. Eur. Urol..

[4] Huguet J. (2013). Follow-up after radical cystectomy based on patterns of tumour recurrence and its risk factors. Actas Urol. Esp..

[5] Ghoneim M.A., Abdel-Latif M., el-Mekresh M., Abol-Enein H., Mosbah A., Ashamallah A., el-Baz M.A. (2008). Radical cystectomy for carcinoma of the bladder: 2720 consecutive cases 5 years later. J. Urol..

[6] Sjodahl G., Abrahamsson J., Holmsten K., Bernardo C., Chebil G., Eriksson P., Johansson I., Kollberg P., Lindh C., Lovgren K. (2022). Different Responses to Neoadjuvant Chemotherapy in Urothelial Carcinoma Molecular Subtypes. Eur. Urol..

[7] von der Maase H., Hansen S.W., Roberts J.T., Dogliotti L., Oliver T., Moore M.J., Bodrogi I., Albers P., Knuth A., Lippert C.M. (2000). Gemcitabine and cisplatin versus methotrexate, vinblastine, doxorubicin, and cisplatin in advanced or metastatic bladder cancer: Results of a large, randomized, multinational, multicenter, phase III study. J. Clin. Oncol..

[8] Lancaster M.A., Knoblich J.A. (2014). Organogenesis in a dish: Modeling development and disease using organoid technologies. Science.

[9] Sato T., Stange D.E., Ferrante M., Vries R.G., Van Es J.H., Van den Brink S., Van Houdt W.J., Pronk A., Van Gorp J., Siersema P.D. (2011). Long-term expansion of epithelial organoids from human colon, adenoma, adenocarcinoma, and Barrett’s epithelium. Gastroenterology.

[10] Wei Y., Amend B., Todenhöfer T., Lipke N., Aicher W.K., Fend F., Stenzl A., Harland N. (2022). Urinary Tract Tumor Organoids Reveal Eminent Differences in Drug Sensitivities When Compared to 2-Dimensional Culture Systems. Int. J. Mol. Sci..

[11] Clevers H. (2016). Modeling Development and Disease with Organoids. Cell.

[12] Kim E., Choi S., Kang B., Kong J., Kim Y., Yoon W.H., Lee H.R., Kim S., Kim H.M., Lee H. (2020). Creation of bladder assembloids mimicking tissue regeneration and cancer. Nature.

[13] Lee S.H., Hu W., Matulay J.T., Silva M.V., Owczarek T.B., Kim K., Chua C.W., Barlow L.J., Kandoth C., Williams A.B. (2018). Tumor Evolution and Drug Response in Patient-Derived Organoid Models of Bladder Cancer. Cell.

[14] Drost J., Clevers H. (2018). Organoids in cancer research. Nat. Rev. Cancer.

[15] Pasch C.A., Favreau P.F., Yueh A.E., Babiarz C.P., Gillette A.A., Sharick J.T., Karim M.R., Nickel K.P., DeZeeuw A.K., Sprackling C.M. (2019). Patient-Derived Cancer Organoid Cultures to Predict Sensitivity to Chemotherapy and Radiation. Clin. Cancer Res..

[16] Hill S.J., Decker B., Roberts E.A., Horowitz N.S., Muto M.G., Worley M.J., Feltmate C.M., Nucci M.R., Swisher E.M., Nguyen H. (2018). Prediction of DNA repair inhibitor response in short-term patient-derived ovarian cancer organoids. Cancer Discov..

[17] Minoli M., Cantore T., Hanhart D., Kiener M., Fedrizzi T., La Manna F., Karkampouna S., Chouvardas P., Genitsch V., Rodriguez-Calero A. (2023). Bladder cancer organoids as a functional system to model different disease stages and therapy response. Nat. Commun..

[18] Yao Y., Xu X., Yang L., Zhu J., Wan J., Shen L., Xia F., Fu G., Deng Y., Pan M. (2020). Patient-derived organoids predict chemoradiation responses of locally advanced rectal cancer. Cell Stem Cell.

[19] Pauli C., Hopkins B.D., Prandi D., Shaw R., Fedrizzi T., Sboner A., Sailer V., Augello M., Puca L., Rosati R. (2017). Personalized In Vitro and In Vivo Cancer Models to Guide Precision MedicinePersonalized Cancer Models to Guide Precision Medicine. Cancer Discov..

[20] Medle B., Sjödahl G., Eriksson P., Liedberg F., Höglund M., Bernardo C. (2022). Patient-derived bladder cancer organoid models in tumor biology and drug testing: A systematic review. Cancers.

[21] Grossman H.B., Soloway M., Messing E., Katz G., Stein B., Kassabian V., Shen Y. (2006). Surveillance for recurrent bladder cancer using a point-of-care proteomic assay. JAMA.

[22] Veeramachaneni R., Nordberg M.L., Shi R., Herrera G.A., Turbat-Herrera E.A. (2003). Evaluation of fluorescence in situ hybridization as an ancillary tool to urine cytology in diagnosing urothelial carcinoma. Diagn. Cytopathol..

[23] Farrow G.M. (1990). Urine cytology in the detection of bladder cancer: A critical approach. J. Occup. Med..

[24] Elbadawy M., Usui T., Mori T., Tsunedomi R., Hazama S., Nabeta R., Uchide T., Fukushima R., Yoshida T., Shibutani M. (2019). Establishment of a novel experimental model for muscle-invasive bladder cancer using a dog bladder cancer organoid culture. Cancer Sci..

[25] Geng R., Harland N., Montes-Mojarro I.A., Fend F., Aicher W.K., Stenzl A., Amend B. (2022). CD24: A Marker for an Extended Expansion Potential of Urothelial Cancer Cell Organoids In Vitro?. Int. J. Mol. Sci..

[26] Mullenders J., de Jongh E., Brousali A., Roosen M., Blom J.P.A., Begthel H., Korving J., Jonges T., Kranenburg O., Meijer R. (2019). Mouse and human urothelial cancer organoids: A tool for bladder cancer research. Proc. Natl. Acad. Sci. USA.

[27] Yu L., Li Z., Mei H., Li W., Chen D., Liu L., Zhang Z., Sun Y., Song F., Chen W. (2021). Patient-derived organoids of bladder cancer recapitulate antigen expression profiles and serve as a personal evaluation model for CAR-T cells in vitro. Clin. Transl. Immunol..

[28] Jiang J., Ulbright T.M., Younger C., Sanchez K., Bostwick D.G., Koch M.O., Eble J.N., Cheng L. (2001). Cytokeratin 7 and cytokeratin 20 in primary urinary bladder carcinoma and matched lymph node metastasis. Arch. Pathol. Lab. Med..

[29] Ravanini J.N., Assato A.K., Wakamatsu A., Alves V.A.F. (2021). Combined use of immunohistochemical markers of basal and luminal subtypes in urothelial carcinoma of the bladder: Association with clinicopathological features and outcomes. Clinics.

[30] Agarwal H., Babu S., Rana C., Kumar M., Singhai A., Shankhwar S.N., Singh V., Sinha R.J. (2019). Diagnostic utility of GATA3 immunohistochemical expression in urothelial carcinoma. Indian J. Pathol. Microbiol..

[31] Abugomaa A., Elbadawy M., Yamawaki H., Usui T., Sasaki K. (2020). Emerging Roles of Cancer Stem Cells in Bladder Cancer Progression, Tumorigenesis, and Resistance to Chemotherapy: A Potential Therapeutic Target for Bladder Cancer. Cells.

