# Supplementary material for: A Protocol for Organoids from the Urine of Bladder Cancer Patients
Source: Cells. 2023 Aug 31;12(17):2188. doi: 10.3390/cells12172188 (PMC10487035; doi:10.3390/cells12172188)
Supplement: Supplementary file 1 [file cells-12-02188-s001.zip › Figure S1.pdf]

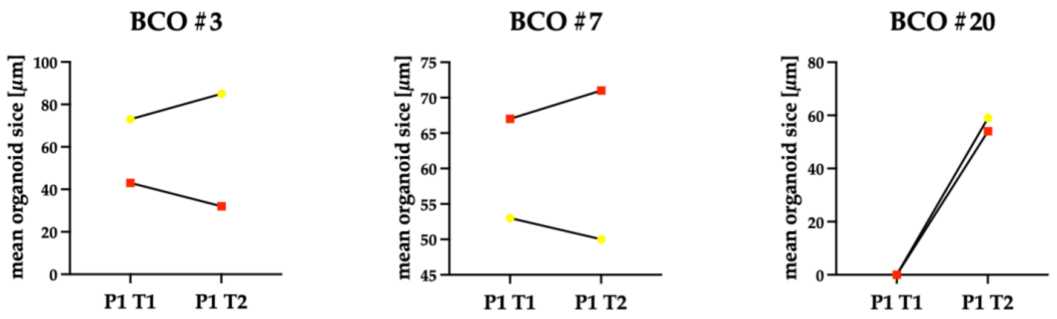


**Figure S1.** Exemplarily shown, mean growth of 3 paired cultures of urin- (indicated in yellow) and tissue-derived (indicated in red) BCOs of the same patient displaying individual growth patterns within a time span of 5 days in the 1st passage. Abbreviations: BCO, bladder cancer organoid; µm, micrometer.
