# Supplementary material for: A Protocol for Organoids from the Urine of Bladder Cancer Patients
Source: Cells. 2023 Aug 31;12(17):2188. doi: 10.3390/cells12172188 (PMC10487035; doi:10.3390/cells12172188)
Supplement: Supplementary file 1 [file cells-12-02188-s001.zip › Figure S2.pdf]

uBCO#13

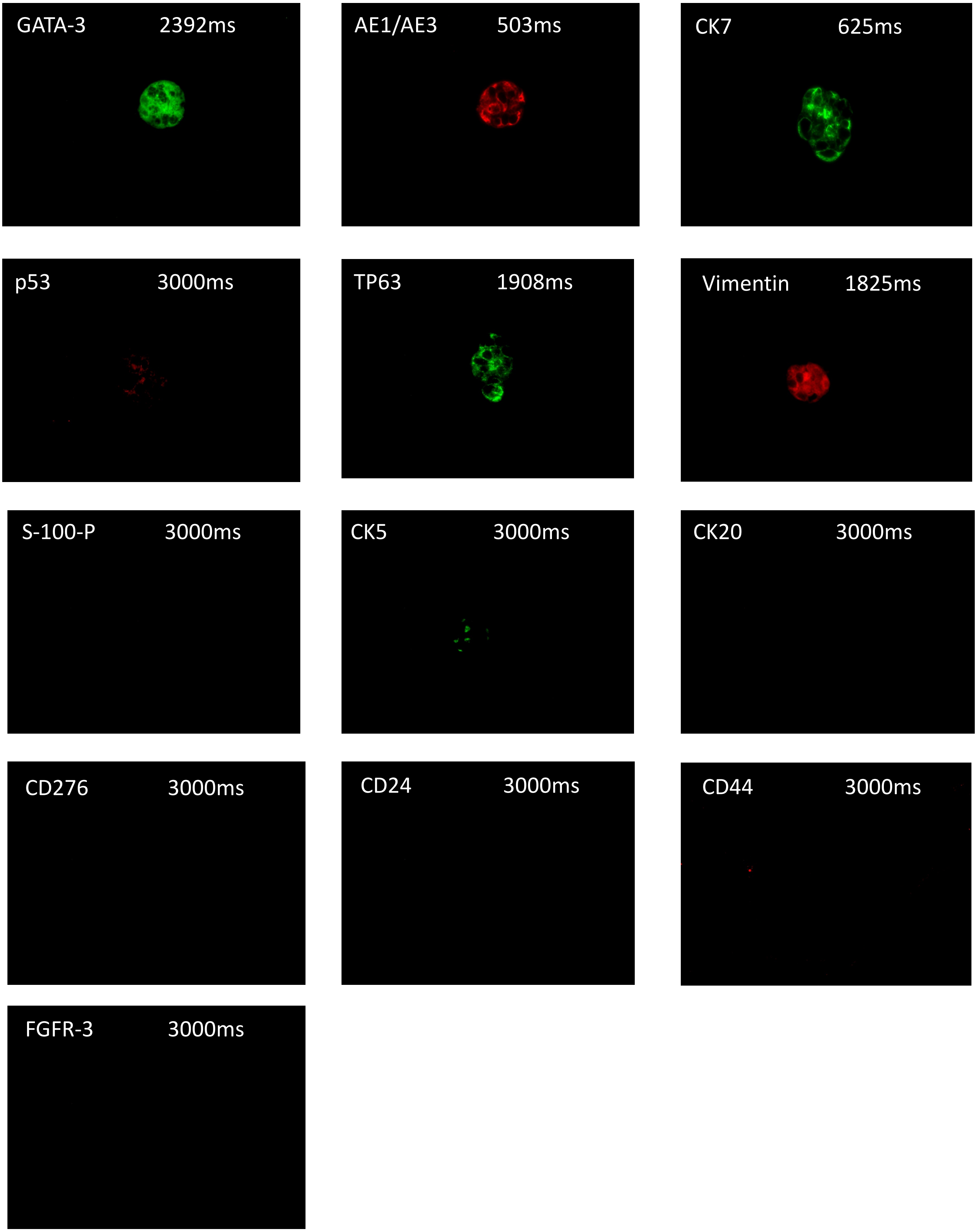

GATA-3 2392ms
AE1/AE3 503ms
CK7 625ms
p53 3000ms
TP63 1908ms
Vimentin 1825ms
S-100-P 3000ms
CK5 3000ms
CK20 3000ms
CD276 3000ms
CD24 3000ms
CD44 3000ms
FGFR-3 3000ms

uBCO#15

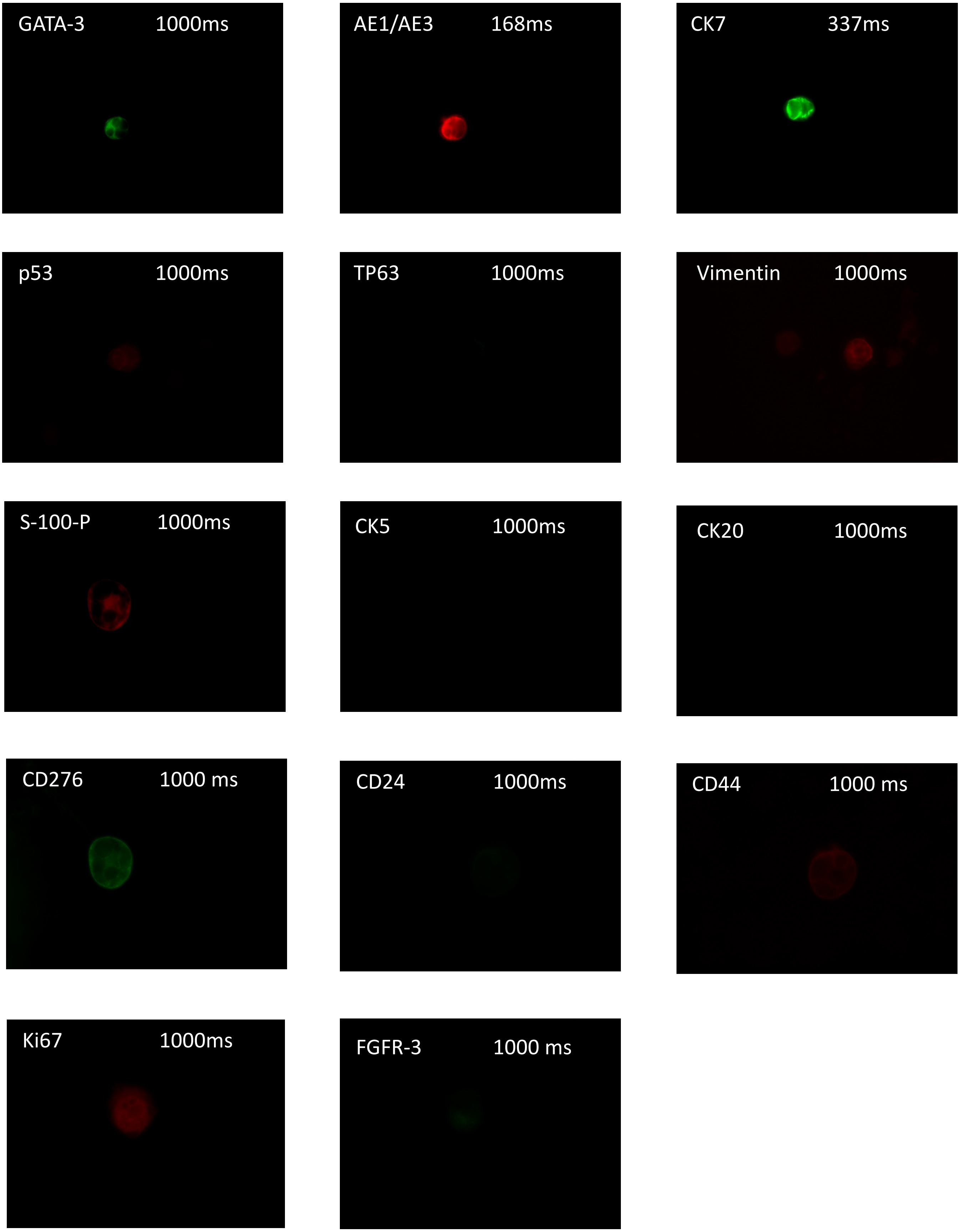
GATA-3
1000ms
AE1/AE3
168ms
CK7
337ms
p53
1000ms
TP63
1000ms
Vimentin
1000ms
S-100-P
1000ms
CK5
1000ms
CK20
1000ms
CD276
1000 ms
CD24
1000ms
CD44
1000 ms
Ki67
1000ms
FGFR-3
1000 ms

uBCO#19

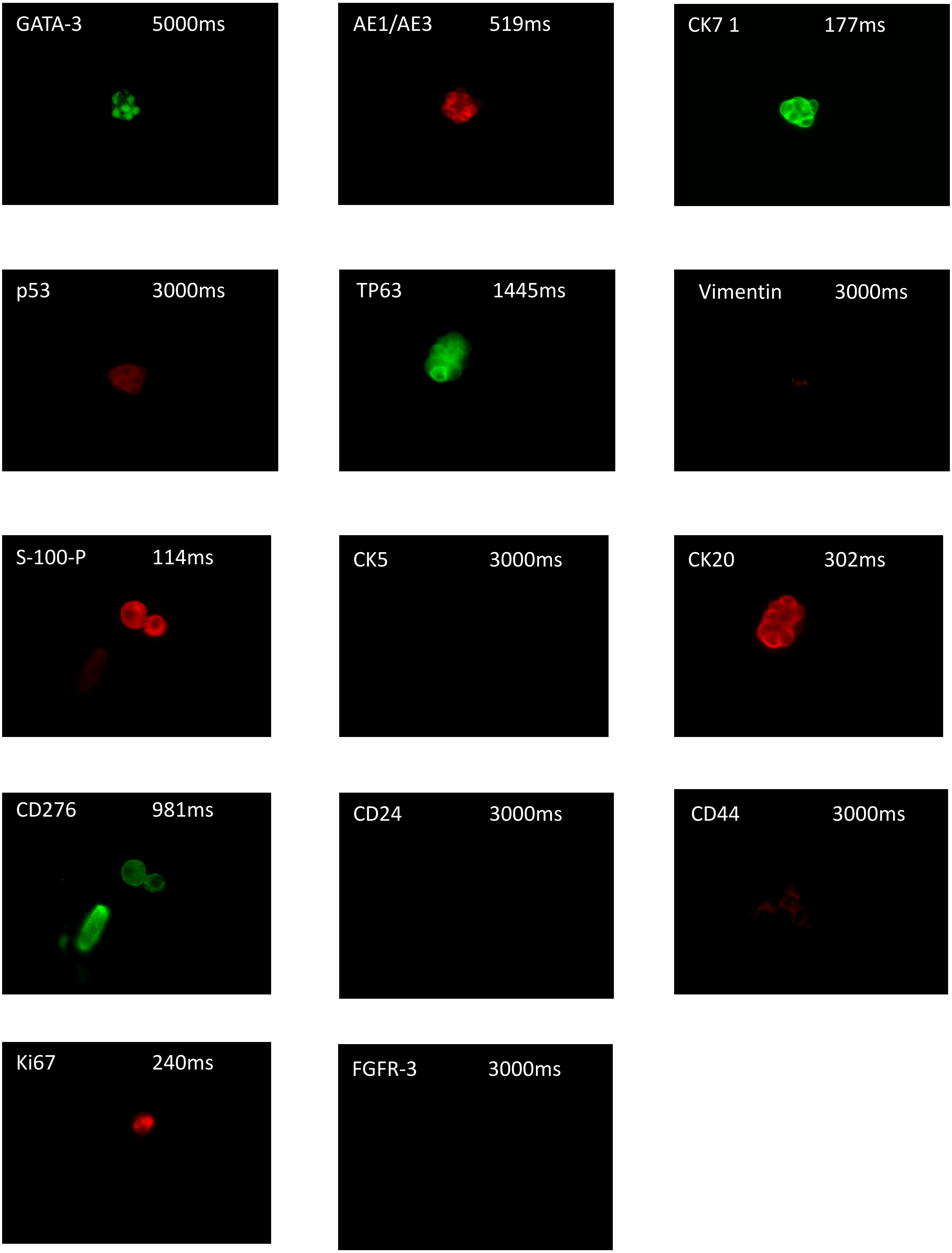
GATA-3 5000ms
AE1/AE3 519ms
CK7 1 177ms
p53 3000ms
TP63 1445ms
Vimentin 3000ms
S-100-P 114ms
CK5 3000ms
CK20 302ms
CD276 981ms
CD24 3000ms
CD44 3000ms
Ki67 240ms
FGFR-3 3000ms

uBCO#22

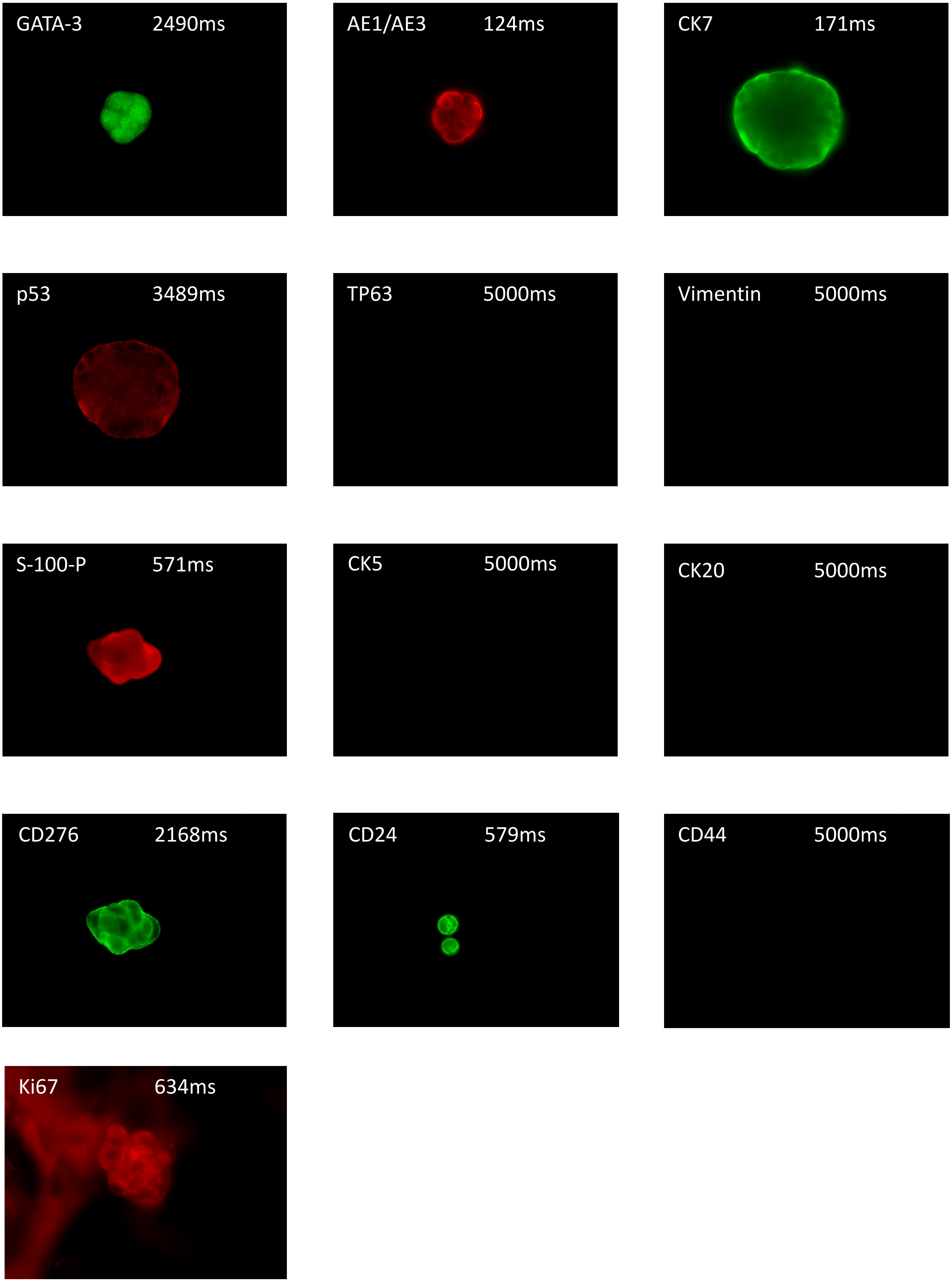
GATA-3 2490ms
AE1/AE3 124ms
CK7 171ms
p53 3489ms
TP63 5000ms
Vimentin 5000ms
S-100-P 571ms
CK5 5000ms
CK20 5000ms
CD276 2168ms
CD24 579ms
CD44 5000ms
Ki67 634ms

uBCO#28

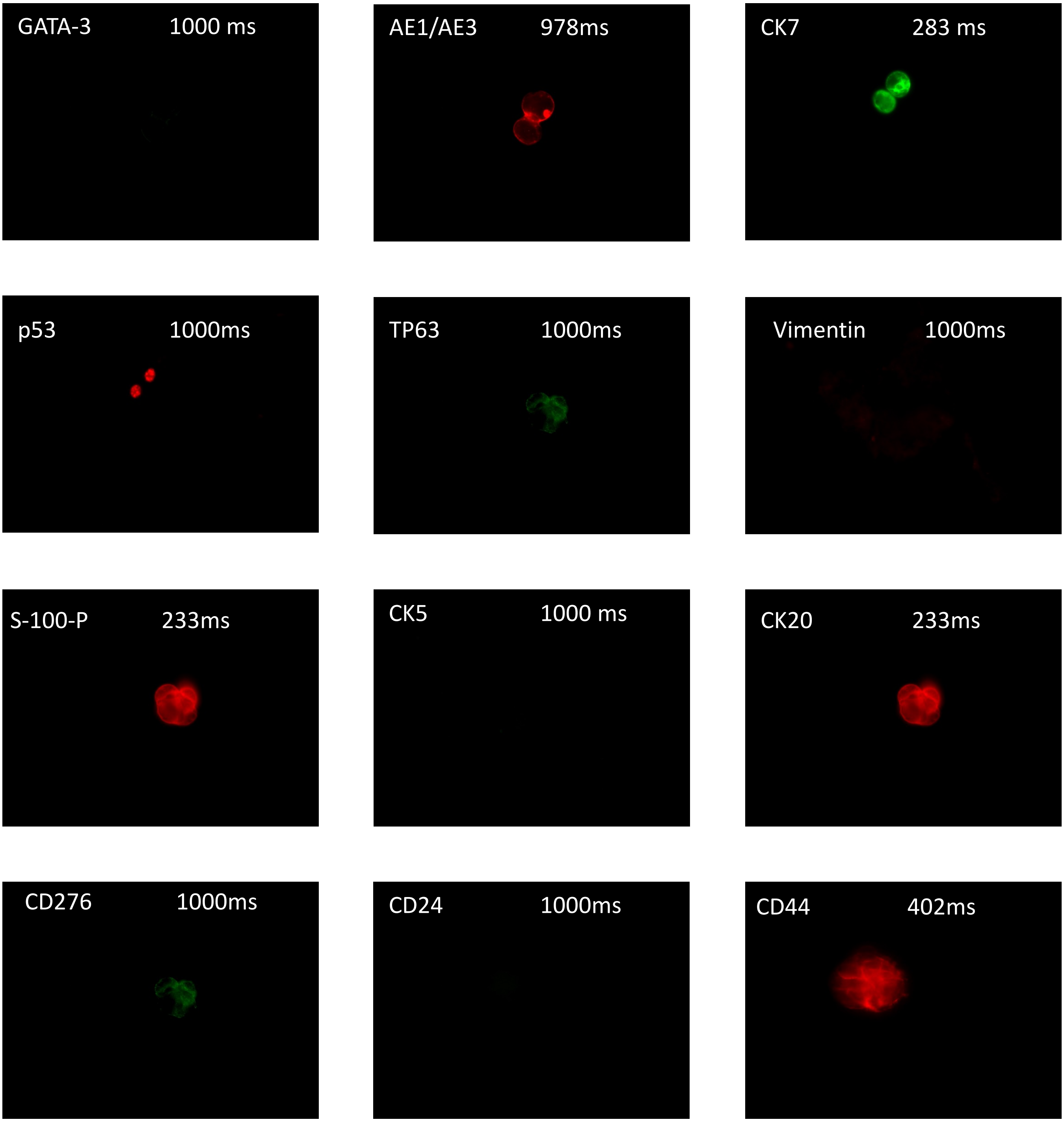

Figure S2. Immunofluorescence characterization of the uBCOs in chamber slides and their corresponding exposure times indicated in ms. Abbreviations: GATA, glutamyl amino transferase A; AE, anti-epithelial; p, protein; TP, tumor protein; CK, cytokeratin; CD, cluster of differentiation; uBCO, urine-derived bladder cancer organoid, Ki, Kiel; FGFR, fibroblast growth factor receptor; uBCO, urine-derived bladder cancer organoid; ms, millisecond.
