# Supplementary material for: A Protocol for Organoids from the Urine of Bladder Cancer Patients
Source: Cells. 2023 Aug 31;12(17):2188. doi: 10.3390/cells12172188 (PMC10487035; doi:10.3390/cells12172188)
Supplement: Supplementary file 1 [file cells-12-02188-s001.zip › Table S1.pdf]

Table S1. Antibodies employed for chamber slides and paraffin sections. Abbreviations: cs, chamber slides; ps, paraffin section

Antibody	Company	Dilution cs	Dilution ps	clone	Caltalog number
Alexa488	Jackson Immuno Research, UK	1:1000	1:1000	-	115-165-166
DAPI	Sigma Aldrich, USA	1:1000	1:1000	-	SigmaD8417 10mg/28,4ml H2O
Cy3	Jackson Immuno Research, UK	1:1000	1:1000	-	111-545-045
rb.Anti-GATA-3	Abcam, UK	1:100	1:50	IgG, polyclonal	Ab106625
rb.Anti-CK7	Abcam, UK	1:300	1:100	IgG, monoclonal EPR1619Y	Ab68459
rb.Anti-CK5	BioLegend, USA	1:200	1:50	Mainly IgG, polyclonal poly19055	905504/100µg
rb.Anti-CD276	Abcam, UK	1:100	1:100	IgG, polyclonal	Ab226256
rb.Anti-TP63	Invitrogen, Thermo Fischer, USA	1:100	1:50	IgG, recombinant, monoclonal 10H7L17	703809
rb.Anti-CD24	Abcam, UK	1:100	1:50	IgG, EPR19925	Ab202073
rb.Anti-FGFR-3	Abcam, UK	1:100	-	IgG1, monoclonal EPR2281(3)	Ab155960
ms.Anti-AE1/AE3	Merck, DE	1:200	1:100	IgG1, monoclonaler clone AE1/AE3	Mab3412
ms.Anti-p53	Invitrogen, Thermo Fischer, USA	1:100	1:50	IgG1, monoclonal, PAb 240	MA5-15244
ms.Anti-Vimentin	Becton Dickinson, USA	1:100	1:50	IgG1, monoclonal, RV202	550513
ms.Anti-S-100-P	Becton Dickinson, USA	1:100	1:100	IgG1, monoclonal, 16/S100P	610306
ms.Anti-CK20	Merck, DE	1:100	1:50	IgMk, monoclonaler Klon XQ1	Mabt845-25UG
ms.Anti-FGFR-3	Invitrogen, Thermo Fischer, USA	-	1:50	IgG1, monoclonal OTI1B10	MA5-26493
ms.Anti-CD44	Abcam, UK	1:200	1:50	IgG1, MEM-263	Ab9524
ms.Anti-Ki67	Biomol-Arigo,	1:100	1:100	IgG1, monoclonal, ARG57562	Arg57562
